# Effects of SGM Education for Undergraduate Medical Students in a Canadian Context

**DOI:** 10.1007/s40670-023-01831-x

**Published:** 2023-07-07

**Authors:** Nessika Karsenti, Jason Chambers, Aldo Espinosa

**Affiliations:** 1 Schulich School of Medicine and Dentistry, Western University, 1151 Richmond St., London, ON Canada; 2 Department of Anesthesia, Schulich School of Medicine and Dentistry, Western University, London, ON Canada

**Keywords:** Sexual and gender minority (SGM), Medical education, Canada

## Abstract

Medical schools have been striving to equip students with the tools and skills needed to serve patients from the LGBTQ + community, also called the Sexual and Gender Minority (SGM) community. This study aims to assess student comfort with providing care, and faculty knowledge and preparedness in delivering SGM-centered education at our home institution. We conducted two mixed-methods surveys, one geared towards medical students across four years of study and one towards medicine faculty. Each survey collected first demographic information about participants, then used a validated tool to assess knowledge of the SGM community. The qualitative component of both surveys then consisted of a needs assessment to determine what students felt should be changed about their curriculum, and what faculty felt should change about their training to deliver this curriculum. We received 26 student responses from all 4 years of study and 35 faculty responses from a variety of medical specialties. Difference in knowledge assessment scores was not statistically significant across both cohorts. Most students felt overall comfortable providing care for sexual minority individuals, and faculty similarly felt comfortable teaching, but data showcases that perceived comfort is higher among the student cohort. We propose that students are acquiring knowledge and comfort with providing for SGM individuals from sources outside their curriculum, and that additional training of faculty is vital to ensure students not doing this independent learning do not fall through the cracks.

**Supplementary Information:**

The online version contains supplementary material available at 10.1007/s40670-023-01831-x.

## Introduction

In Canada, it is estimated that approximately 1% of the population identifies as lesbian, gay, bisexual, trans, or otherwise a member of the Sexual and Gender Minority (SGM) community [1]. However, there is a wide body of literature documenting the disparities felt by this population when navigating the medical system [2]. As budding physicians, today’s medical students are increasingly aware that a portion of their patients will be SGM populations, no matter their area of practice. A push towards Competency-Based Medical Education (CBME) in medical education institutions has provided an opportune platform for more SGM content in curricula to help students address this need [3]. There has, however, been considerable criticism of the degree to which these topics are taught, from SGM community members, faculty, and medical students alike [4–7], and suggestions have been made for establishing a national standard for education [4, 8].

Current research into the impact and efficacy of medical school curricula pertaining to the SGM community in Canada is lacking; the last national evaluation of curricular content was published in 2011 [9]. One study evaluating students’ perception of their curriculum in Canada was conducted in 2017, and found that 85% of students at the University of Ottawa want to have further education on SGM communities [4]. Only one evaluation of the efficacy of a curriculum has been conducted in Canada in the last 10 years to our knowledge. The Northern Ontario School of Medicine conducted a curriculum evaluation in 2018 and found that their new curriculum was effective in increasing student knowledge in SGM issues irrespective of their previous level of knowledge, and that this improvement was greater when faculty training was greater [10].There is clearly a paucity of current and Canadian research into this topic.

As CBME was only recently adopted into the curriculum at our institution in 2020 [11], we sought to understand the impact that this curriculum change has had on understanding of, and confidence in, providing for SGM individuals. This paper describes a mixed methods analysis of two surveys, one geared towards students across four years of medical training at our institution, and one distributed to faculty members. This survey was originally distributed as part of an internal quality improvement initiative, and as such aimed to answer questions relevant to the first two steps of the Kern model of curriculum development [12], “problem identification and general needs assessment” and “targeted needs assessment”, by collecting perspectives from both students and faculty. The goal of this initiative was to assess knowledge about SGM community members at our institution and determine how students and faculty perceive the adequacy of their curriculum in preparing new students to provide for SGM community members. Additionally, self-identified members of the SGM community who received the surveys were invited to share additional information about their experiences within the health system and our institution. By comparing data within and between both survey results, as well as across 4 years of medical training, we assessed student and faculty knowledge of SGM individuals and gained a better understanding both of how students perceived this portion of their curriculum and how equipped faculty feel to teach these topics.

## Methods

### Student Survey

We developed the survey distributed to students through numerous discussions with all three authors and a review of questions obtained from past papers that have conducted similar studies [13, 14]. Initial questions first ask for demographic information, such as age, gender identity, sexual identity, race, and socioeconomic status (see Appendix 1). Each team member scrutinized each question to ensure the information was needed to ensure a representative sample and worded so as to not introduce additional bias into the survey. Then, we adapted a standardized tool, the Sex Education and Knowledge about Homosexuality Questionnaire (SEKHQ) [15], a set of 32 true or false questions for assessing knowledge of sexual minority individuals for use in a more modern setting. This questionnaire has been previously used for research of this kind numerous times and in varying contexts [16, 17], and is therefore a well-established research tool. We modified questions wherever possible to include gender minorities, which were not adequately reflected in the original tool (see Appendix 1). Finally, we included a set of questions developed by the research team and inspired by previous studies of this nature [13, 14] to assess perceived adequacy of the curriculum and opinions for potential improvements. These were a mix of multiple choice, Likert-type scales, multi-select, and short answer questions. Students who self-identified as SGM minorities in the demographics section were then presented with three additional open-ended questions designed to further understand their experience navigating the medical community as SGM individuals. All student survey questions can be found in Appendix 1. Although we use “SGM” throughout this paper, our survey questions used the term “LGBTQ” instead, as this was felt to be a more familiar term to our target respondents.

The survey was electronically built using the 2021 version of Qualtrics XM (Qualtrics, Provo, UT), an online survey building and distribution platform. We distributed this survey using an anonymous link that was sent by email to all medical students in their first, second, third, or fourth year of training using the medical institution’s student listserv per institutional policies. Our institution has 170 medical students per year of study. Given the timing of CBME implementation at our institution, first, second and third year students have been taught using this new model and fourth year students have experienced the prior curriculum. The survey was distributed at the same time to all three years of study. A reminder email about this survey was automatically sent two weeks after the initial recruitment email. We kept the survey open for one month from the initial distribution date.

### Faculty Survey

We built the faculty survey similarly to the student survey, using consensus from the entire study team. The same demographic information and the same modified standardized knowledge assessment tool were included, followed by similar questions pertaining to curriculum delivery to assess lecturer’s perceived comfort with teaching medical students about SGM issues. SGM self-identifying faculty were also invited to answer open-ended questions about their experiences navigating the medical field. We also built this survey using Qualtrics, distributed an anonymous link to the survey using the institutional faculty listserv, comprised of all faculty that engage in medical student education including clinicians and academically-trained individuals who teach both in a classroom and hospital setting, and left it open for one month for data collection. All faculty survey questions can be found in Appendix 1.

### Quantitative Data analysis

Quantitative data was analyzed using GraphPad Prism 9 (GraphPad Software Inc, San Diego, California) software. For ease of analysis and due to the small sample size for most categories included in multiple choice questions, we re-grouped demographic data into binomial categories and used Fisher’s exact test to compare student and faculty demographics and ensure no statistical difference between both samples. We regrouped demographic data as follows: Sex assigned at birth (male or female), Gender identity (cis or non-cis), Sexual orientation (heterosexual or non-heterosexual), Faith (declares a faith or no faith/atheist), and Race (Caucasian vs person of color).

The knowledge assessment portion of the surveys was automatically graded by Qualtrics as per the answer key in Appendix 1, giving each participant a score out of 32. We compared mean scores between faculty and students using a Mann–Whitney test. We compared scores to demographic data as well, to determine whether any demographic out-performed others on this assessment. The same binomial groupings described above were again used, and we performed Mann–Whitney tests to compare knowledge assessment scores and sex assigned at birth, gender identity, sexual orientation, faith and race for both student and faculty groups. We created a composite between faculty and student cohorts so that the binomial groups of sex assigned at birth and sexual orientation could be compared using Mann–Whitney tests as a correction for unequal demographic distributions in these groups.

We analyzed Likert-type questions as ordinal variables and provide a mode and median with range as descriptive statistics by converting Likert scale responses from 1–5 or 1–7 as required (depending on the number of options in the scale itself), with 1 corresponding to the lowest or worst category and 5 or 7 corresponding to the highest or best. When comparing data from two Likert-type scales with equal numbers of responses in both, we used a Kendall tau B to analyze for associations between data sets. When sample sizes were unequal, we used Fischer’s exact test instead [18].

### Qualitative Data Analysis

We compiled short answer questions from students and faculty into a document separate from the rest of the survey questions and tagged each response with the main theme described. We then refined themes to a few repeating categories that help describe overall student and faculty responses to each question [19]. We then used these themes and associated quotes to reinforce conclusions drawn from the quantitative data, as the qualitative data set sample size was insufficient for drawing conclusions on its own.

## Results

### Survey Respondents

We received a total of 26 complete responses to the student survey, from the following classes: 11.5% (n = 3) first year students (class of 2024), 60.8% (n = 14) second year students (class of 2023), 15.4% (n = 4) third year students (class of 2022) and 19.2% (n = 5) fourth year students (class of 2021). The average age of student respondents was 25 (standard deviation 2.04). All respondents disclosed their gender and sexual orientation in this survey. While 92.3% (n = 24) of student respondents identified as cisgender (with the remaining two students identifying as non-binary and gender non-conforming), only 46.1% of respondents self-identified as straight (n = 12), with the remaining being gay (7.40%, n = 2), lesbian (7.40%, n = 2), bisexual (29.6%, n = 8), pansexual (3.70, n = 1), or asexual (3.70%, n = 1). A total of 26.9% (n = 7) of respondents had achieved Master’s level of training prior to beginning medical school. More information about the demographics of survey respondents can be found in Table 1.

**Table 1:** Demographics of student and faculty survey respondents

	Students (n = 26)	Faculty (n = 35)	Comparison
Age	Mean: 25, range: 23–29	Under 45: 9 45–65: 20 Over 65: 6	Not compared—different age groups targeted by definition
Sex assigned at birth	Female: 20 Male: 6	Female: 12 Male: 23	**p = 0.0009**
Gender identity	Cisgender: 24 Non-binary: 1 Gender non-conforming: 1	Cisgender: 35	p = 0.5752
Sexual orientation	Straight: 12 Gay: 2 Lesbian: 2 Bisexual: 8 Pansexual: 1 Asexual: 1	Straight: 27 Gay: 5 Lesbian: 1 Bisexual: 2	**p = 0.0312**
Race	Caucasian: 17 POC: 9	Caucasian: 25 POC: 7 Did not disclose: 3	p = 0.3861
Religion	Has a faith: 10 No faith/atheist: 16	Has a faith: 16 No faith/atheist:18	p = 0.7957
Graduation year	2021: 6 2022: 4 2023: 14 2024: 3	N/A	N/A
Previous education	Bachelor’s: 19 Master’s: 7	N/A	N/A

*N/A* Not asked in this respective survey, and not compared

Significant p values are in **bold**

Similarly, we received a total of 35 complete survey responses from faculty members from various fields of study. 57.1% (n = 20) respondents were between the ages of 45–65, representing the largest age bracket; 25.7% (n = 9) were below the age of 45 and 17.1% (n = 6) were above 65. With regard to gender identity, 100.0% (n = 35) of faculty identified as cisgender. In contrast to student respondents, 77.1% (n = 27) of faculty self-identified as straight, while 14.3% (n = 5) identified as gay, 2.86% (n = 1) lesbian, and 5.71% (n = 2) bisexual. More information about the demographics of faculty respondents can be found in Table 1.

We also compared student and faculty demographics to assess whether a significant difference in sample makeup is present. The student cohort had a higher proportional number of women (n = 20 females and n = 6 males for students, n = 12 females and n = 23 males for faculty) and this difference proved to be significant (p = 0.0017). Similarly, the student cohort had a greater number of non-heterosexual respondents (n = 14 non-heterosexual students, n = 12 heterosexual students, n = 8 non-heterosexual faculty, n = 27 heterosexual faculty) and this also proved significantly different (p = 0.0166). Other than gender assigned at birth and sexual orientation, the makeup of each cohort was similar (see Table 1).

### Knowledge Assessment

A total of 26 student participants completed the knowledge assessment. The mean score among students was 20.34 ± 5.19 (median 21.5, range 2–27) out of 32. There was no statistically significant difference in scores between people assigned female and those assigned male at birth (p = 0.1013), those who are heterosexual and those who are not (p = 0.2969), those who follow a faith and those who don’t (p = 0.5028), and those who identified as Caucasian and those who identified as a person of color (p = 0.8618). Similarly, 35 faculty members completed the knowledge assessment, with a mean score of 20.8 ± 4.157 (median score 21, range 10–27) out of 32. There also was no statistically significant difference in scores between people assigned female and those assigned male at birth (p = 0.6475), those who are heterosexual and those who are not (p = 0.1288), those who follow a faith and those who don’t (p = 0.7381), and those who identified as Caucasian and those who identified as a person of color (p = 0.1634). Neither group could be compared across gender identities, as the number of non-cis participants was too small in both groups (n = 0 for faculty, n = 2 for students). There was no statistically significant difference in knowledge assessment scores between faculty and students (p = 0.9623).

When comparing the faculty and student cohorts we can observe that they are similar in demographics apart from gender assigned at birth and sexual orientation. To allow for the comparison of knowledge assessment scores between these cohorts with these demographic difference, we created a student/faculty composite and tested knowledge assessment scores between male assigned at birth and female assigned at birth, and heterosexual vs. non-heterosexual, where we found that both of these divisions were not significantly different (composite heterosexual vs. non-heterosexual had medians of 21 and 22.5 respectively, p = 0.0871 and composite of female vs. male had medians of 22.0 and 20.50 respectively, p = 0.1814) (Table 2).

**Table 2:** Knowledge assessment scores for faculty and students, total and by graduation year

	**Year 1** (n = 3)	**Year 2** (n = 14)	**Year 3** (n = 4)	**Year 4** (n = 5)	**All students** (n = 26)	**All faculty** (n = 35
Minimum	18	11	19	2	2	10
Maximum	23	27	25	23	27	27
Median	20	22.5	22.5	19	21.5	21
Mean (Std Dev)	20.3 (2.5)	21.2 (4.5)	22.2 (2.5)	16.4 (8.23)	20.3 (5.2)	20.8 (4.1)

### Student Familiarity and Comfort with SGM Patients and Issues

When asked to rate their familiarity with concepts relevant to SGM communities prior to medical school, students rated their familiarity highest at “very knowledgeable” for “LGBTQ vocabulary” (mode = 4, median = 4, range = 2–5), “appropriate use of pronouns” (mode = 4, median = 4, range = 2–5), and “the impact of prejudice and discrimination on health” (mode = 4, median = 3, range = 1–5). Lowest rated familiarity was for “what being two-spirit entails” (mode = 1, median = 1, range = 1 to 4), “LGBTQ history” (mode = 2, median = 2, range = 1–4) and “differences in health needs between LGBTQ and non-LGBTQ individuals” (mode = 2, median = 2, range = 1–5). Finally, student responses were mixed with regard to familiarity with “what being transgender entails” (mode = 3, median = 3, range = 1–5).

Overall, students reported increased knowledge of and familiarity with SGM-specific terminology and concepts after starting medical school (Table 3). When comparing familiarity of concepts worded identically before and after medical school, students perceived a statistically significant increase in their familiarity across all 7 metrics, although mode and median did not increase for most (see Table 3 for more information). Students were asked for an additional 5 metrics to rate familiarity of during medical school and overall had moderate familiarity with all five of (mode = 3, median = 3 and range = 2–5 for all) “issues faced by LGBTQ individuals in general”, “issues faced by LGBTQ individuals seeking care”, “differences in health care needs between LGBTQ and non-LGBTQ individuals”, “taking an LGBTQ-sensitive sexual history”, and “the process involved with medically transitioning”.

**Table 3:** Past and current familiarity with concepts from students and faculty

	Before medical school	During medical school	Statistical difference (pre vs post)	Faculty	Statistical difference (student current vs faculty)
LGBTQ vocabulary – the definition of gay, lesbian, bisexual, etc	Mode: 4 Median: 4 Range: 2–5	Mode: 4 Median: 4 Range: 3–5	**p = 4.92E-05**	Mode: 3 Median: 3 Range: 1–5	**p = 2.24E-04**
Appropriate use of pronouns	Mode: 4 Median: 4 Range: 2–5	Mode: 4 Median: 4 Range: 2–5	**p = 9.53E-07**	Mode: 3 Median: 3 Range: 1–5	p = 0.060
LGBTQ history	Mode: 2 Median: 2 Range: 1–4	Mode: 2 Median: 2 Range: 1–4	**p = 1.19E-06**	Mode: 2 Median: 2 Range: 1–5	p = 0.183
Differences in health needs between LGBTQ and non-LGBTQ individuals	Mode: 2 Median: 2 Range: 1/5	Mode: 3 Median: 3 Range: 2–5	**p = 1.92E-05**	Mode: 3 Median: 3 Range: 1–5	p = 0.144
The impact of prejudice and discrimination on health	Mode: 4 Median: 3 Range: 1–5	Mode: 4 Median: 4 Range: 2–5	**p = 9.54E-07**	Mode: 3 Median: 3 Range: 1–5	**p = 0.029**
What being transgender entails	Mode: 3 Median: 3 Range: 1–5	Mode: 3 Median: 3 Range: 2–5	**p = 1.43E-06**	Mode: 3 Median: 3 Range: 1–5	p = 0.049
What being two-spirit entails	Mode: 1 Median: 1 Range: 1–4	Mode: 3 Median: 2 Range: 1–4	**p = 1.91E-06**	Mode: 1 Median: 1.5 Range: 1–5	**p = 0.016**
Issues faced by LGBTQ individuals in general	Not asked	Mode: 3 Median: 3 Range: 2–5	NA	Not asked	NA
Issues faced by LGBTQ individuals seeking healthcare	Not asked	Mode: 3 Median: 3 Range: 2–5	NA	Not asked	NA
Differences in health care needs between LGBTQ individuals and non-LGBTQ individuals	Not asked	Mode: 3 Median: 3 Range: 2–5	NA	Not asked	NA
Taking a LGBTQ-sensitive sexual history	Not asked	Mode: 3 Median: 3 Range: 2–5	NA	Not asked	NA
The process involved with medically transitioning, including the involvement of a psychologist, drugs and surgeries	Not asked	Mode: 3 Median: 3 Range: 2–5	NA	Not asked	NA

This survey was administered to all students at the same time and asked recall questions pertaining to their level of knowledge before medical school and perceived knowledge now, during medical school. Likert scales for this question was a 5-point scale, ranging from “not knowledgeable at all” (given a 1) to “very knowledgeable” (given a 5)

Statistically significant p values are in **bold**

Not asked: This question was not asked to this particular demographic or in this particular part of the survey. *NA* Because this question was not asked, there is no comparison to be made and therefore no p value to report

Students were asked to identify concepts that were formally taught to them before and during medical school. Four of these concepts were repeated in both sets of questions, and students reported more teaching on them prior to medical school on average—for instance, 22.22% (n = 6) of students reported being formally taught “the use of appropriate vocabulary for referring to LGBTQ individuals” prior to medical school and only 14.81% (n = 4) of students reported being formally taught this during medical school. Further comparison can be found in Table 4.

**Table 4:** Rates of endorsement of formal teaching of various topics, students and faculty

Concepts formally taught	Students: prior to medical school	Students: during medical school	Faculty: at any time in academic career
The use of appropriate vocabulary for referring to LGBTQ individuals	48.1% n = 13	55.55% n = 15	14.29% n = 5
The use of appropriate pronouns for referring to trans, intersex, or gender non-conforming individuals	40.74% n = 11	40.74% n = 11	5.71% n = 2
LGBTQ history	0% n = 0	0% n = 0	5.71% n = 2
Differences in health needs between LGBTQ and non-LGBTQ individuals	22.22% n = 6	40.74% n = 11	11.43% n = 4
The impact of prejudice and discrimination on health	51.85% n = 14	N/A	31.43% n = 11
What being transgender entails	51.85% n = 14	N/A	11.43% n = 4
What being two-spirit entails	11.11% n = 3	N/A	5.71% n = 2
Issues faced by LGBTQ individuals in general	N/A	37.04% n = 10	N/A
Issues faced by LGBTQ individuals seeking health care	N/A	66.66% n = 18	N/A
Taking a LGBTQ-sensitive sexual history	N/A	48.15% n = 13	N/A
The process involved with medically transitioning, including the involvement of a psychologist, drugs, and surgeries	N/A	66.66% n = 18	N/A

*N/A* This question was not asked to this particular demographic or in this particular part of the survey

Furthermore, students on average responded “very comfortable” on a series of questions relating to their comfort in providing care to members of the SGM population in general and to transgender patients (mode = 4, median = 4 and range = 2–5 for both) but responded only “moderately comfortable” for interactions with or providing care to intersex patients (mode = 3, median = 3, range = 2–5). To assess how the university helped students develop this comfort, we asked them to identify opportunities for interactions with SGM patients that were offered throughout their time in medical school. 25.92% (n = 7) of students identified “interactions with LGBTQ patients” and “practice interviewing with standardized patients” as an offered opportunity; 29.62% (n = 8) identified “meeting LGBTQ physicians” and only 3.70% (n = 1) identified “shadowing or involvement in clinics that focus on LGBTQ health”.

When questioned about potential improvements to the program, students responded on average either “somewhat agree” “agree” or “strongly agree” to 12 topics or concepts they feel should be implemented in future iterations of their curriculum (see Table 5). We also asked students to identify modes of learning they felt would be most efficient for learning these topics. From most to least effective, students identified “opportunities to take sexual histories with standardized patients” (77.77%, n = 21), “panels with LGBTQ individuals” (74.07%, n = 20), “small group sessions” (62.96%, n = 17), “lectures” (55.55%, n = 15), “online learning modules” (29.63%, n = 8), and one participant wrote in the answer “small group sessions with 2SLGBTQ individuals”.

**Table 5:** Perceptions of topics to be included in future iterations of the curriculum

	Students	Faculty	P value
The use of appropriate vocabulary for referring to LGBTQ individuals	Mode: 7 Median: 5 Range: 1–7	Mode: 6 Median: 6 Range: 1–7	p = 0.182619
The use of appropriate pronouns for referring to trans, intersex, or gender non-conforming individuals	Mode: 7 Median: 5 Range: 1–7	Mode: 6 Median: 6 Range: 1–7	p = 0.174167
LGBTQ history	Mode: 6 Median: 6 Range: 4–7	Mode: 4 Median: 5 Range: 1–7	p = 0.142089
Differences in health needs between LGBTQ and non-LGBTQ individuals	Mode: 7 Median: 7 Range: 3–7	Mode: 7 Median: 6.5 Range: 3–7	p = 0.94102
The impact of prejudice and discrimination on health	Mode: 7 Median: 6 Range: 2–7	Mode: 7 Median: 7 Range: 4–7	p = 0.055523
What being transgender entails	Mode: 6 Median: 6 Range: 3–7	Mode: 6 Median: 6 Range: 1–7	p = 0.093331
What being two-spirit entails	Mode: 7 Median: 6 Range: 3–7	Mode: 7 Median: 6 Range: 1–7	p = 0.276411
Issues faced by LGBTQ individuals in general	Mode: 5 Median: 5 Range: 3–7	Mode: 6 Median: 6 Range: 3–7	p = 0.57797
Issues faced by LGBTQ individuals seeking healthcare	Mode: 7 Median: 7 Range: 3–7	Mode: 7 Median: 6 Range: 3–7	p = 0.526948
Differences in health care needs between LGBTQ individuals and non-LGBTQ individuals	Mode: 7 Median: 7 Range: 4–7	Mode: 7 Median: 6 Range: 3–7	p = 0.676539
Taking a LGBTQ-sensitive sexual history	Mode: 7 Median: 7 Range: 4–7	Mode: 7 Median: 6 Range: 1–7	p = 0.125674
The process involved with medically transitioning, including the involvement of a psychologist, drugs and surgeries	Mode: 7 Median: 6 Range: 3–7	Mode: 7 Median: 6 Range: 1–7	p = 0.734004

Likert scales for this question was a 7-point scale, ranging from “strongly disagree” (given a 1) to “strongly agree” (given a 7)

None of the p values are significant

### Current Teaching Gaps

Faculty were first asked to evaluate their familiarity with the same 7 concepts that were presented to students. On average, faculty rated their current knowledge as “moderate” for “LGBTQ vocabulary” (mode = 3, median = 3, range = 1–5), “appropriate use of pronouns” (mode = 3, median = 3, range = 1–5), “differences in health needs between LGBTQ and non-LGBTQ individuals” (mode = 3, median = 3, range = 1–5), “what being transgender entails” (mode = 3, median = 3, range = 1–5), and “the impact of prejudice and discrimination on health” (mode = 3, median = 3, range = 1–5). Lowest rated familiarity was for “LGBTQ history” (mode = 2, median = 2, range = 1–5) and “what being two-spirit entails” (mode = 1, median = 1.5, range = 1 to 5). Faculty members were also asked to identify which of these concepts had been formally taught to them at some point in their training, with response rates varying from 31.42% (n = 11) endorsing being taught “the impact of prejudice and discrimination on health” to only 5.71% (n = 2) endorsing being taught “the use of appropriate pronouns”, “LGBTQ history” and “what being two-spirit entails” (see Table 4). Additionally, 80% (n = 28) of faculty members specified this training was not part of their official institutionally mandated training when getting hired (14.28% (n = 5) stated they were unsure if they had formal SGM-related teaching as part of the hiring process).

When asked about topics that should be taught as part of medical school curriculum for undergraduate medical students, faculty responded on average either “agree” or “strongly agree” to a list of 12 topics, barring “LGBTQ history”, which they rated lower (mode = 4, median = 5, range = 1–7) (see Table 5).

Finally, faculty were asked about their perception of how safe SGM-identifying students were. Average responses indicate faculty feel that gay, lesbian and bisexual students are “moderately safe” (mode = 3, median = 3, range = 1–4), transgender men and women and men who acted feminine were “slightly unsafe” (mode = 2, median = 2, range = 1–4), as were women who acted masculine (mode = 2, median = 3, range = 1–4). Two spirit individuals were perceived to also be “slightly unsafe” (mode = 2, median = 2.5, range = 1–4). When asked whether faculty members felt it was partly their responsibility to ensure student safety, responses were overall in strong agreement, with 2.86% (n = 1) responding “disagree”, 0% (n = 0) responding “neither agree nor disagree”, 11.43% (n = 4) responding “agree” and the remaining 85.7% (n = 30) responding “strongly agree”. Faculty were then asked to rank potential methods for improving the learning environment of SGM students and overall ranked “faculty sensitivity training”, “including LGBTQ matters in the curriculum” and “the opportunity to meet LGBTQ physicians” as “useful” (mode = 4, median = 4, range = 1–5) and “promoting anti-harassment and anti-discrimination policies” as “extremely useful” (mode = 5, median = 4, range = 1–5).

### Comparing Student Faculty Responses

Comparing student and faculty level of familiarity across 7 SGM-related concepts showed that on average, students perceive themselves to have a higher level of familiarity or knowledge than faculty. This comparison was statistically significant in three instances, “LGBTQ vocabulary” (p = 0.0002), “the impact of prejudice and discrimination on health” (p = 0.0290), and “what being two-spirit entails (p = 0.0157). Categories that were not statistically significant include “what being transgender entails” (p = 0.0487), “the use of appropriate pronouns” (p = 0.0060), “LGBTQ history (p = 0.1833), and “differences in health needs between queer and non-queer patients” (p = 0.1443) (see Table 3). Students and faculty were of very similar opinions regarding concepts that should be better taught by medical schools and comparing them proved to be statistically insignificant across all 12 metrics (see Table 5).

### Safety and Security in Medicine

Nine student respondents and six faculty respondents elected to answer short-answer questions pertaining to their experience identifying as a member of the SGM community in medicine and their overall perceptions of the medical school curriculum. Through grouping responses under particular themes (see Table 6), students and faculty alike discussed incidences of discrimination, microaggression, and stereotyping by other faculty members or students. In two instances, this discrimination had a religious component, and in two others, hypersexuality of SGM individuals was discussed. Students have described encounters with their own physicians where assumptions were made about their sexuality, and where a physician denied care as they were not familiar enough with a set of medication to prescribe it. When discussing good portrayal of the SGM community in medical curricula, respondents gave examples of cases with standardized patients. These examples, when given by students, were always met with a caveat about either “being too rushed” or “gay/lesbian representation with [..] no transgender representation”. This same lack of representation was echoed by faculty as well when asked about weak points in the curriculum. In addition to the above three themes, students have identified one additional theme that faculty did not: a lack of faculty knowledge. This difference between the two groups could be due to sampling bias within the faculty group, as faculty with a vested interest in providing this education would be both more interested in answering our survey and more knowledgeable on the topic.

**Table 6:** Qualitative themes obtained from open-answer questions in both surveys

Theme	Example quote—students	Example quote—faculty
Discrimination	Someone yelled "God hates gays" at me	Othering is a persistent and endemic culture in London and the longer I am here the more it is apparent. It really took my partner pointing it out and he now refuses to attend events with other faculty as he feels it much more acutely than I do
Microaggressions	Not sure if this counts, but I often get the assumption that if I'm sexually active there's a chance I could be pregnant (I'm dating a woman right now and there is no chance I'm pregnant). I've stopped answering yes when asked because I don't always want to come out to my doctor, especially one who assumes I'm in a heterosexual relationship	I passed as a straight married physician for 25 years and then came out and people were very supportive and I faced no open hostility; however, when passing as straight one heard the jokes or the innuendos about out gay colleagues
Stereotyping within the curriculum	I appreciate the representation but it always feels forced and pretty stereotyped/tokenized. Plus, I've honestly only seen gay/lesbian representation, nothing significant of transgender people or other minority genders and sexualities	That particular case (within the curriculum) is outdated with respect to current availability of rapid HIV testing, and the need to test MSMs (men who have sex with men)
Paucity of faculty knowledge	We had a cishet lady teach us about trans health while constantly confusing the difference between sex and gender	N/A

Four main themes that emerged from analysis of the qualitative portion of both surveys; this table compares example quotes for each theme from the student and faculty surveys

*N/A* no quote about this topic in this group

## Discussion

### Appropriateness of Sample

Our sample size of students and faculty is small, and the two cohorts differed in that student respondents were more female, and of more non-heterosexual orientation. Additionally, the proportion of queer-identifying students and faculty who responded to our survey (35.48%) is greater than the national average (1%) [1], although the proportion of medical providers who are queer as compared to the national average is not known. This is most likely attributed to our recruitment method, as individuals were approached over email and could choose whether to participate in this survey; it stands to reason that SGM-identifying people are more likely to choose to be involved in research that could impact their own community. While this is an important source of bias to consider when interpreting our data, this also serves to enrich the qualitative data obtained from this survey by obtaining more examples and perspectives from the community these topics try to serve. It is difficult to generalize student and faculty knowledge of the SGM community given this small sample size; however, this limitation can also be a strength of our methodology as SGM-identifying students and faculty may be more likely to take note of and want to communicate perceived issues within their curriculum, thus enriching our needs assessment data. In addition, it is important to note that a significant portion of student respondents are in their preclinical years, with very little exposure to patient interaction; therefore, their perceived knowledge or comfort could change once this is tested through caring for an SGM patient during their clinical training. Finally the small sample of fourth year students (n = 5) makes it impossible to compare students who have not received teaching through the CBME model to students who have; however, these survey results still shed important light on how the current CBME model is impacting students' comfort. Importantly, the implementation of CBME at this institution did not come with associated changes to faculty training; data collected from faculty therefore has no control group to compare to.

### Students as Teachers

Comparing rates of self-perceived familiarity and knowledge of key concepts before and after medical school among students yields interesting results. First, on average students rated an increased level of knowledge throughout medical school compared to prior, which was statistically significant in all metrics. However, students endorsed being formally taught these same concepts at higher rates prior to medical school compared to during it. Students also feel their curriculum needs to better teach these concepts across the board. This seems to indicate that, although students are improving in their knowledge and familiarity of concepts important to treating LGBTQ populations, this increased knowledge is not coming from their formal curriculum but are rather being taught in other spaces. In fact, this is echoed by a student who notes in a short answer question that “A student-led presentation by the LGBTQ2 + club that explained gender and sexual identity in a far more accurate and less harmful way than the actual curricular content”. It would be an interesting avenue of further research to determine where students feel they learn the most about issues faced by queer patients (social media, other student-led initiatives, etc.). Furthermore, students rated their familiarity with these concepts higher than faculty did on average, and this was statistically significant for three of seven metrics. There were no metrics where faculty rated their knowledge higher than students. It is difficult to ascertain whether students are truly more knowledgeable than faculty given the small sample size and that both groups tested equally well on the knowledge assessment. However, this could indicate that students are truly more knowledgeable than their faculty counterparts in ways that are not captured by the knowledge assessment, which mostly asks questions about sexual identities, omitting gender identities before our modifications. It could also indicate faculty may need additional support or training in how they share their knowledge with students (for example, sensitivity training). Importantly, over 80% of our faculty respondents stated they had no formal training on SGM topics prior to teaching. This is further endorsed by students who commented that “our designated lecture actually gave inaccurate, outdated and at times harmful misinformation about sexual and gender identity”. Another student gave a more salient example; “We had a cishet lady teach us about trans health while constantly confusing the difference between sex and gender – it’s like she knew the concepts but didn’t have the vocab.” One faculty member also remarks that “There are examples of generational differences in the portrayal of the LGBTQ + community with opportunity for bidirectional sharing.”, indicating that faculty are aware that students can play the role of teacher for these topics.

It is important to again note the potential bias introduced by the number of students who responded to this survey and identify as SGM—these students can be increasingly motivated to learn about these topics outside the classroom. However, this still provides an interesting avenue for further research—do students who are less motivated to seek out these topics fall through the cracks if their faculty are not prepared to teach them?

### The Hidden Curriculum

Paul et al. argue that congruence of the formal curriculum (what we assess here), informal and hidden curriculum must be achieved for proper cultural competence training [20]. In this instance, the hidden curriculum would represent the interactions that students have with faculty beyond the formal learning objectives; it also represents the kind of material that is presented to students about SGM individuals when the focus is not on their sexual or gender identity [21, 22].

An example of the hidden curriculum is one where case examples of SGM-identifying patients is used [21]. This theme was brought up several times by our student respondents, with both positive examples—“the patient was a lesbian, with a business, but if I remember correctly, was distant with family members (potentially due to sexual orientation)”—and negative ones such as “Bisexual man cheating on his wife” or “the "hard part" turned out to be the fact that the patient was in a relationship with a woman and was not out to her parents. It did not feel great that this was the thing we had to "figure out" to help the patient, because it felt like it was making being 2SLGBTQ + out to be difficult and it was reducing the patient to that part of her identity.” Clinical cases that stereotype SGM-identifying patients are well-documented. Turbes et al. comment that in an analysis of over 900 clinical cases, sexual orientation was usually only mentioned in the context of HIV risk assessment [21]**.** One of the students who responded to this survey comments “it seems that whenever there is a LGBTQ patient in case-based learning, there is an HIV concern, or something like that. I think this is dangerous because it may further stigmatize gay men with regard to HIV/AIDS.” it is clear the hidden curriculum has incongruencies with the formal curriculum, and this is one of the major issues that students perceive with the curriculum overall.

The impact of the hidden curriculum extends beyond the selection of clinical cases and into interactions between students and faculty within and beyond the learning environment. For instance, when discussing microaggressions experienced in school, one student comments “a preceptor during PCCM interviewing [a clinical methods course] stated that we must insist on always taking an in-depth sexual history of our bi and lesbian patients because "they are all sexually active" (even if they are single and state that)”. The hypersexuality of queer individuals is a common misconception and harmful stereotype [23] that in this case, leads a doctor to not believe a patient’s sexual history. Teaching that all “bi and lesbian patients” are sexually active is not part of session objectives, or formal curriculum; having faculty teach this to students is an example of hidden curriculum where a harmful stereotype has been perpetrated. It is also a great example of an instance where additionally training faculty participating in clinical methods courses could be beneficial.

Importantly, faculty do not endorse a lot of formal teaching of such issues at any point in their academic career, and only two faculty respondents indicated that their formal teaching was a mandatory part of their hiring process at the institution. While not all faculty hired by a medical institution can be expected to teach SGM-related content, they may all interact with learners while treating SGM-identifying patients or otherwise be discussing SGM-related care with students. Improving the instruction or training given to faculty may improve learning outcomes for medical students [24], and this is an important area of future research.

## Recommendations for Improvement

Results from this survey have the potential to guide further curricular developments at our institution. Based on student ratings of their own knowledge, the biggest curricular gaps appear to be in areas of LGBTQ history, differences in health care needs between SGM individuals and non-SGM individuals, and taking a sensitive history, among others (see Table 3). Additionally, students and faculty alike agree that the most lacking topics within the curriculum are these same differences in health needs and taking this history (see Table 5). Students have identified the following modalities as their preferred ways for curricular content: the opportunity to take a sexual history with a standardized patient and panels with SGM individuals [25, 26]. In addition, the discrepancy in comfort with these concepts between students and faculty do indicate that additional training of faculty could be beneficial for aligning the formal and hidden curriculum. These are some preliminary avenues for improvement indicated by our faculty and students that is echoed by a large body of literature that discusses improvements in curricular standards [27].

## Conclusion

Our results demonstrate that overall, students are just as knowledgeable about SGM issues as their faculty are. Given that our sampling method encouraged individuals with a vested interest in the issue to respond (as demonstrated by the higher proportion of SGM individuals in the student cohort), it is quite likely that they represent a proportion of the student body with a better understanding of LGBTQ healthcare provision. A deeper look, however, shows that students are most likely not learning this knowledge from their faculty, and this vested interest may be the driving force behind their increased perceived comfort. Therefore, standardizing and improving the curriculum is imperative to ensure students who are less passionate about this topic do not fall through the cracks. This should start with educating faculty to be key knowledge keepers and providers for their students. While adopting a CBME model for curricular implementation at our institution had the goal of better preparing all students to be comfortable providing care to SGM patients in Canada, there was no change made to faculty development with the implementation of this model. Further training of faculty would help fill gaps and allow a better congruence between the formal and hidden curriculum, which students and researchers have indicated to be one of the most important barriers to training health care providers who are safe for SGM individuals. Our research demonstrates that students and faculty ideals are in alignment with this goal.

## Supplementary Information

Below is the link to the electronic supplementary material.

Supplementary file1 (DOCX 31 KB)

## Data Availability

The datasets generated during the current study are not publicly available as the small sample size precludes the ability to maintain subject confidentiality.
