# Supplementary material for: Effects of SGM Education for Undergraduate Medical Students in a Canadian Context
Source: Med Sci Educ. 2023 Jul 7;33(4):813–24. doi: 10.1007/s40670-023-01831-x (PMC10403457; doi:10.1007/s40670-023-01831-x)
Supplement: Supplementary file 1 — Supplementary file1 (DOCX 31 KB) [file 40670_2023_1831_MOESM1_ESM.docx]

**Appendix 1**

Appendix 1 - Questions included in surveys for both students and faculty. Only a subset of questions in this survey are presented in this paper, but all questions presented to participants are included in this appendix. Answers to the true or false “knowledge assessment” section are included in brackets at the beginning of the statement [(9)](https://www.zotero.org/google-docs/?JAoCHx). For Likert-type questions, each option is listed with a weighting in square brackets that was used for data analysis. This weighting was not presented to survey participants [(14)](https://www.zotero.org/google-docs/?9FrYqJ).

| Section | Question | Question type | Student survey, faculty survey, or both |
| --- | --- | --- | --- |
| Demographics | Age | Numerical | Both |
|  | Gender assigned at birth   - Male - Female - Would rather not disclose | Single selection | Both |
|  | Gender identity   - Cisgender man - Cisgender woman - Transgender man - Transgender woman - Non-binary - Gender non-conforming - Two-spirit - Other (please specify) - Would rather not disclose | Single selection | Both |
|  | Sexual orientation   - Straight - Gay - Lesbian - Bisexual - Pansexual - Asexual - Other (please specify) - Would rather not disclose | Single selection | Both |
|  | Population of town or city in which the participant grew up   - 0-999 - 1,000-9,999 - 10,000-99,999 - +100,000 | Single selection | Students only |
|  | Race or ethnicity | Short answer | Both |
|  | Faith or religion | Short answer | Both |
|  | Parents annual combined income   - 9,999 or less - 10,000-49,999 - 50,000-99,999 - +100,000 - Don’t know or would rather not disclose | Single selection | Students only |
|  | Anticipated graduation year from medical school   - 2024 - 2023 - 2022 - 2021 | Single select | Students only |
|  | Highest level of education achieved prior to medical school   - Bachelor’s - Master’s - PhD | Single select | Students only |
|  | Area of study prior to medical school | Short answer | Students only |
|  | Area of work/study within the Faculty of Medicine | Short answer | Faculty only |
| Knowledge assessment [(9)](https://www.zotero.org/google-docs/?hH0nio) | (F) Approximately 25-30% of adolescent boys have a gay experience during their teenage years | True, false or unsure | Both |
|  | (F) A majority of gay/lesbian people were seduced in adolescence by a person of the same sex, usually several years older | True, false or unsure | Both |
|  | (T) Approximately 6-11% of adolescent girls have a lesbian experience during their teenage years | True, false or unsure | Both |
|  | (T) Sexual orientation is usually well-established by adolescence | True, false or unsure | Both |
|  | (T) Gay/lesbian people usually disclose their sexual identity to a friend before they tell a parent | True, false or unsure | Both |
|  | (F) A gay/lesbian person’s gender identity does not agree with his/her biological sex | True, false or unsure | Both |
|  | (F) If children are raised by openly same-gender parents, the likelihood that they themselves will develop a gay/lesbian orientation is greater than if they were raised by heterosexual parents | True, false or unsure | Both |
|  | (T) Gay men and lesbian women have an increased incidence of anxiety and depression compared to heterosexual men and women | True, false or unsure | Both |
|  | (F) Gay/lesbian people place more importance on the physical attractiveness of their dating partners than do heterosexual people. | True, false or unsure | Both |
|  | (T) The experience of love is similar for all people regardless of sexual orientation | True, false or unsure | Both |
|  | (T) Gay male couples are likely to have the most permissive attitudes about sexual activity outside of a committed relationship compared to lesbian couples and heterosexual couples | True, false or unsure | Both |
|  | (T) In some cultures, it is normal practice for boys to have sex with their same-gender during adolescence. | True, false or unsure | Both |
|  | (F) In the world as a whole, the most common mode of transmission of the HIV virus is through gay male sex | True, false or unsure | Both |
|  | (T) Testosterone is the hormone responsible for the growth of pubic hair on girls | True, false or unsure | Both |
|  | (T) Boys’ breasts typically grow during puberty | True, false or unsure | Both |
|  | (F) Research supports the notion that sex education offered in schools increases the amount of sexual activity amongst adolescents | True, false or unsure | Both |
|  | (F) In the last 25 years there has been an increase in the number of gay men and lesbian women. | True, false or unsure | Both |
|  | (F) Most gay men and lesbian women want to be heterosexual | True, false or unsure | Both |
|  | (F) Most gay/lesbian or trans people want to encourage or entice others into a gay/lesbian or trans lifestyle | True, false or unsure | Both |
|  | (T) Heterosexual teachers, more often than gay/lesbian teachers, seduce their students or sexually exploit them | True, false or unsure | Both |
|  | (F) Greece and Rome fell because of gay/lesbian people | True, false or unsure | Both |
|  | (F) Heterosexuals generally have a stronger sex drive than do gay/lesbian people | True, false or unsure | Both |
|  | (T) About one-half of the population of men and more than one-third of women have had a gay/lesbian experience to the point of orgasm at some time in their lives | True, false or unsure | Both |
|  | (T) There are more gay men than lesbian women | True, false or unsure | Both |
|  | (T) Heterosexual men and women commonly report gay/lesbian fantasies. | True, false or unsure | Both |
|  | (F) If the media portrays being gay or lesbian or trans as positive, this could sway youths into becoming gay/lesbian or trans or desiring a gay/lesbian or trans way of life. | True, false or unsure | Both |
|  | (F) Gay/lesbian people are usually identifiable by their appearance or mannerisms | True, false or unsure | Both |
|  | (F) Gay/lesbian or trans people do not make good role models for children and could do psychological harm to children with whom they interact as well as interfere with the normal sexual development of children | True, false or unsure | Both |
|  | (T) Gay men are more likely to be victims of violent crime than the general public | True, false or unsure | Both |
|  | (F) Animals do not engage in sexual activity with same gender partners | True, false or unsure | Both |
|  | (F) Historically, almost every culture has evidenced widespread intolerance towards gay/lesbian people, viewing them as “sick” or as “sinners” | True, false or unsure | Both |
|  | (T) Heterosexual men tend to express more hostile attitudes towards gay/lesbian people than do heterosexual women | True, false or unsure | Both |
| Knowledge prior to medical school | Please select all terms or concepts that you were formally taught as part of your academic education (for students:prior to medical school).   - The use of appropriate vocabulary for referring to LGBTQ individuals - The use of appropriate pronouns for referring to trans, intersex, or gender non-conforming individuals - LGBTQ history - Differences in health needs between LGBTQ and non-LGBTQ individuals - The impact of prejudice and discrimination on health - What being transgender entails - What being two-spirit entails | Multi-select | Both |
|  | Was this formal teaching part of faculty training upon being hired as a faculty member at the University of Western Ontario?   - Yes - Unsure - No | Single-select | Faculty only |
|  | Please rate your familiarity with the following terms or concepts prior to beginning medical school   - The use of appropriate vocabulary for referring to LGBTQ individuals - The use of appropriate pronouns for referring to trans, intersex, or gender non-conforming individuals - LGBTQ history - Differences in health needs between LGBTQ and non-LGBTQ individuals - The impact of prejudice and discrimination on health - What being transgender entails - What being two-spirit entails | Likert scale  - Not knowledgeable at all [1]  -Slightly knowledgeable [2]  -Moderately knowledgeable [3]  -Very knowledgeable [4]  - Extremely knowledgeable [5] | Students only |
| Current familiarity and comfort | Please rate your CURRENT familiarity with the following terms or concepts:   - The use of appropriate vocabulary for referring to LGBTQ individuals - The use of appropriate pronouns for referring to trans, intersex, or gender non-conforming individuals - LGBTQ history - Differences in health needs between LGBTQ and non-LGBTQ individuals - The impact of prejudice and discrimination on health - What being transgender entails - What being two-spirit entails   The following were included for students only:   - Issues faced by LGBTQ individuals in general - Issues faced by LGBTQ individuals seeking healthcare - Differences in health care needs between LGBTQ individuals and non-LGBTQ individuals - Taking a LGBTQ-sensitive sexual history - The process involved with medically transitioning, including the involvement of a psychologist, drugs and surgeries | Likert scale  - Not knowledgeable at all [1]  -Slightly knowledgeable [2]  -Moderately knowledgeable [3]  -Very knowledgeable [4]  - Extremely knowledgeable [5] | Both (last 5 for students only) |
|  | Please rate your current comfort level with the following statements   - Interactions with LGBTQ patients - Interactions with transgender patients - Interactions with Intersex patients - Providing medical care to LGBTQ patients - Providing medical care to transgender patients - Providing medical care to intersex patients | Likert scale  -Not comfortable at all [1]  -Slightly comfortable [2]  -Moderately comfortable [3]  -Very comfortable [4]  -Extremely comfortable [5] | Students only |
| Perception of student safety | How safe do you think the following students feel at the Institution?   - Gay - Lesbian - Bisexual - Transgender male - Transgender female - Male who acts feminine - Female who acts masculine - 2-spirit individual | Likert scale  -Not at all safe [1]  -Slightly unsafe [2]  -Moderately safe [3]  -Very safe [4] | Faculty only |
|  | How much do you agree with the following statement? “Teachers and other school personnel have an obligation to ensure a safe and supportive learning environment for gay, lesbian, bisexual and transgender students | Likert scale:  -Strongly disagree [1]  - Disagree [2]  -Neither agree nor disagree [3]  -Agree [4]  -Strongly agree [5] | Faculty only |
|  | How useful would the following efforts be in creating a better learning environment for LGBTQ2 students?   - Faculty sensitivity training - Promoting anti-harrassment and anti-discrimination policies - Including LGBTQ matters in the curriculum - Opportunity to meet LGBTQ physicians | Likert scale:  -Extremely useless [1]  -Useless [2]  -Neither useful nor useless [3]  -Useful [4]  -Extremely useful [5] | Faculty only |
| Curricular content | Did your medial curriculum provide you with formal training on any of the following (please select all that apply)   - Issues faced by LGBTQ individuals in general - Issues faced by LGBTQ individuals seeking health care - LGBTQ history - The use of appropriate vocabulary for referring to LGBTQ individuals - The use of appropriate pronouns for referring to trans, intersex, or gender-non-conforming individuals - Differences in health care needs between LGBTQ individuals and non-LGBTQ individuals - Taking an LGBTQ-sensitive sexual history - The process involved with medically transitioning, including the involvement of a psychologist, drugs, and surgeries | Multi-select | Students only |
|  | Did your medical school offer you the opportunity for (please select all that apply)   - Interactions with LGBTQ patients - Practice interviewing with standardized patients - Opportunity to meet LGBTQ physicians - Shadowing in clinics that focus on LGBTQ health | Multi-select | Students only |
| Perceived curricular gaps | My medical curriculum or education should provide more of   - The use of appropriate vocabulary for referring to LGBTQ individuals - The use of appropriate pronouns for referring to trans, intersex, or gender non-conforming individuals - LGBTQ history - Differences in health needs between LGBTQ and non-LGBTQ individuals - The impact of prejudice and discrimination on health - What being transgender entails - What being two-spirit entails - Issues faced by LGBTQ individuals in general - Issues faced by LGBTQ individuals seeking healthcare - Differences in health care needs between LGBTQ individuals and non-LGBTQ individuals - Taking a LGBTQ-sensitive sexual history - The process involved with medically transitioning, including the involvement of a psychologist, drugs and surgeries | Likert scale  -Strongly disagree [1]  -Disagree [2]  -Somewhat disagree [3]  -Neither agree nor disagree [4]  -Somewhat agree [5]  -Agree [6]  -Strongly agree [7] | Both |
|  | I would best prefer to learn through (please select all that apply)   - Lectures - Small group sessions - Panels with LGBTQ individuals - Online learning - The opportunity to take sexual histories with standardized patients - Other, please specify | Multi-select | Students only |
| For self-identifying SGM students | If you have experienced harassment, discrimination, etc as a result of your gender identity or sexual identity PRIOR to medical school, please describe this or these instances as much as you are comfortable. | Free text | LGBTQ-identifying students only |
|  | If you have experienced harassment, discrimination, etc as a result of your gender identity or sexual identity trying to access health care, please describe this or these instances as much as you are comfortable. | Free text | Both, LGBTQ-identifying only |
|  | Are there examples from your current medical curriculum you think have portrayed the LGBTQ community well? If yes, please describe. | Free text | Both, LGBTQ-identifying only |
|  | Are there examples from your current medical curriculum you think have portrayed the LGBTQ community poorly? If yes, please describe. | Free text | Both, LGBTQ-identifying only |
|  | Do you consent to having the above experiences disclosed to faculty and future students in the context of curriculum renewal? Complete anonymity is assured and you can withdraw this consent at any time.   - Yes - faculty only - Yes - students only - Yes - faculty and students - No - I do not consent to having the above experiences disclosed to faculty or students - I am unsure about my willingness to consent and would like contact the research team to obtain more information | Single select | Both, LGBTQ-identifying only |
